# Peripheral MC1R Activation Modulates Immune Responses and is Neuroprotective in a Mouse Model of Parkinson’s Disease

**DOI:** 10.1007/s11481-023-10094-7

**Published:** 2023-12-19

**Authors:** Pranay Srivastava, Shuhei Nishiyama, Fang Zhou, Sonia H. Lin, Akriti Srivastava, Chienwen Su, Yuehang Xu, Weiyi Peng, Michael Levy, Michael Schwarzschild, Xiqun Chen

**Affiliations:** 1 MassGeneral Institute for Neurodegenerative Disease, Department of Neurology, Massachusetts General Hospital, Harvard Medical School, Boston, USA; 2 Aligning Science Across Parkinson’s (ASAP) Collaborative Research Network, Chevy Chase, MD USA; 3 Department of Biology and Biochemistry, University of Houston, Houston, TX USA

**Keywords:** BBB, LPS, Melanocortin 1 Receptor, MPTP, Parkinson’s Disease, Regulatory T Cells (Tregs)

## Abstract

**Background:**

Melanocortin 1 receptor (*MC1R*) is a key pigmentation gene, and loss-of-function of *MC1R* variants that produce red hair may be associated with Parkinson’s disease (PD). We previously reported compromised dopaminergic neuron survival in *Mc1r* mutant mice and dopaminergic neuroprotective effects of local injection of a MC1R agonist to the brain or a systemically administered MC1R agonist with appreciable central nervous system (CNS) permeability. Beyond melanocytes and dopaminergic neurons, MC1R is expressed in other peripheral tissues and cell types, including immune cells. The present study investigates the impact of NDP-MSH, a synthetic melanocortin receptor (MCR) agonist that does not cross BBB, on the immune system and the nigrostriatal dopaminergic system in mouse model of PD.

**Methods:**

C57BL/6 mice were treated systemically with MPTP.HCl (20 mg/kg) and LPS (1 mg/kg) from day 1 to day 4 and NDP-MSH (400 µg/kg) or vehicle from day 1 to day 12 following which the mice were sacrificed. Peripheral and CNS immune cells were phenotyped and inflammatory markers were measured. The nigrostriatal dopaminergic system was assessed behaviorally, chemically, immunologically, and pathologically. To understand the role of regulatory T cells (Tregs) in this model, CD25 monoclonal antibody was used to deplete CD25 + Tregs.

**Results:**

Systemic NDP-MSH administration significantly attenuated striatal dopamine depletion and nigral dopaminergic neuron loss induced by MPTP + LPS. It improved the behavioral outcomes in the pole test. *Mc1r* mutant mice injected with NDP-MSH in the MPTP and LPS paradigm showed no changes in striatal dopamine levels suggesting that the NDP-MSH acts through the MC1R pathway. Although no NDP-MSH was detected in the brain, peripheral, NDP-MSH attenuated neuroinflammation as observed by diminished microglial activation in the nigral region, along with reduced TNF-α and IL1β levels in the ventral midbrain. Depletion of Tregs was associated with diminished neuroprotective effects of NDP-MSH.

**Conclusions:**

Our study demonstrates that peripherally acting NDP-MSH confers protection on dopaminergic nigrostriatal neurons and reduces hyperactivated microglia. NDP-MSH modulates peripheral immune responses, and Tregs may be involved in the neuroprotective effect of NDP-MSH.

## Background

Melanocortin receptors (MCRs) are a family of five G-protein coupled receptors. Among them, melanocortin 1 receptor (MC1R) is expressed in melanocytes and regulates pigmentation of the skin and hair. Upon binding to its ligand alpha-melanocyte stimulating hormone (α-MSH), MC1R activates the cAMP pathway and facilitates the synthesis of brown/black pigment eumelanin, increasing the ratio of red/blonde pheomelanin to eumelanin (Swope and Abdel-Malek 2018; Wolf Horrell et al. 2016). Red hair and fair skin in people are usually due to loss-of-function *MC1R* variants and are associated with accelerated skin aging, as well as increased melanoma risk (Law et al. 2017; Williams et al. 2011; Wolf Horrell et al. 2016). Red hair and *MC1R* loss-of-function variants have also been reported to be associated with increased risk for Parkinson’s disease (PD), a common neurodegenerative disease that has been consistently linked to melanoma (Ye et al. 2020).

Pathologically, PD is characterized by loss of dopaminergic neurons in the substantia nigra (SN) of the brain and abnormal accumulation and aggregation of alpha-synuclein (αSyn) in the nervous system. Although the etiology of PD is unclear, oxidative stress, mitochondrial dysfunction, neuronal network alteration, and neuroinflammation have been reported to be important contributors (Titova et al. 2017). Additionally, there is mounting evidence that chronic systemic inflammation (Pretorius et al. 2014) with the accompanying dysregulation of circulating inflammatory molecules and the innate immune response, play prominent roles in PD (Kannarkat et al. 2013). It is increasingly appreciated that peripheral, as well as brain inflammation, contribute to the onset and progression of the neurodegenerative processes in PD (Tansey et al. 2022). Previous studies from our group reported expression of MC1R in dopaminergic neurons (Chen et al. 2017a). *Mc1r* mutant mice showed a compromised dopaminergic system with greater susceptibility to PD-associated mitochondrial toxin MPTP (1-methyl-4-phenyl-1,2,3,6-tetrahydropyridine) and αSyn overexpression, whereas locally administered MCR agonist NDP-MSH ([Nle^4^, DPhe^7^]-α-MSH) attenuated αSyn toxicity in brain of naïve mice (Cai et al. 2018, 2022; Chen et al. 2017a). NDP-MSH is a synthetic analog to α-MSH and is chemically more stable than α-MSH. Similar to α-MSH, NDP-MSH can activate all MCRs, though its affinity is highest for MC1R. NDP-MSH is not detectable in the brain after systemic i.p. injection in C57BL/6 mice (Cai et al. 2022). Both α-MSH and NDP-MSH (Langendonk et al. 2015) have additionally been shown to protect in models of other central nerve system (CNS) disorders after systemic administration, including ischemic stroke, spinal cord injury, traumatic brain injury, Alzheimer’s disease (AD), and other neuroinflammation-associated diseases like intracerebral hemorrhage (Giuliani et al. 2014; Leone et al. 2013; Mykicki et al. 2016; Schaible et al. 2013; Wu et al. 2019). The direct site of the NDP-MSH actions in these models was either known to include CNS due to obvious blood brain barrier (BBB) disruption (Mykicki et al. 2016) or not specifically characterized (Giuliani et al. 2014).

In addition to regulating pigmentation and other cellular functions in melanocytes, MC1R is present in immune cells like CD4^+^ T cells, and monocytes (Kadiri et al. 2021; Reynolds et al. 2010; Salazar-Onfray et al. 2002) and involved in modulating immune responses and inflammation (Catania et al. 2004; Salazar-Onfray et al. 2002). Abnormal immune and inflammatory responses have emerged as prominent factors potentially underlying onset and progression of PD. For example, persistent microglia activation has been well characterized. Besides microglia, another population of immune cells implicated in disease pathogenesis consists of monocytes, which also expresses MC1R (Guida et al. 2022). An analysis of myeloid compartment in PD patients revealed migration of peripheral monocytes to the CNS that was in sync with rodent studies (Grozdanov et al. 2014; Harms et al. 2018). Dopaminergic neurons in SN is highly sensitive to pro-inflammatory cytokines like TNF-α and IFNγ in models of PD (Block et al. 2007; Piri et al. 2022; Tansey and Goldberg 2010). Growing evidence also suggests a role of perturbed peripheral immune components and chronic inflammatory cascades in the pathophysiology of PD (Kannarkat et al. 2013). Populations of peripheral lymphoid cells including CD4^+^ helper T cell, and CD8^+^ cytotoxic T cells were altered in PD patients (Bhatia et al. 2021; Lindestam Arlehamn et al. 2020).

Regulatory T cells (Tregs), forming the immunosuppressive T-cell subset, are involved in maintaining immune homeostasis. Dysregulated Tregs can cause increased levels of proinflammatory mediators leading to exacerbated immune responses in PD (Danikowski et al. 2017; Viglietta et al. 2004; Baek et al. 2016; Ciccocioppo et al. 2019; Kosloski et al. 2013; Takahashi and Sakaguchi 2003) and restoration of Treg function has been proposed to have therapeutic implications (Thome et al. 2021). Reynolds et al. demonstrated a perturbed nigrostriatal dopaminergic system associated with dysfunctional Tregs in an αSyn immunization PD model (Reynolds et al. 2010). Expansion of Treg populations is the mechanism behind the protection by α-MSH and NDP-MSH in experimental autoimmune encephalomyelitis (EAE) acting through MC1R expressed on T cells (Mykicki et al. 2016) .

The present study investigates the effects of the systemic melanocortin activator NDP-MSH in the MPTP mouse model of PD, with systemic inflammation induced by lipopolysaccharide (LPS). LPS has been shown to have poor BBB penetrance, especially at lower doses (Banks et al. 2015; Chung et al. 2010) and systemic LPS likely causes neuroinflammation indirectly (Qin et al. 2007). LPS also activates CD4 + cells, monocytes, and neutrophils and significantly increases the circulating cytokines TNF-α, IL-1β, etc. (Henry et al. 2009; Tough et al. 1997). PD patients exhibit an impaired immune response with the contribution of monocytes, CD4 + cells, regulatory T cells, and increased circulatory cytokines (TNF, IFNγ, IL-1β, IL-6, IL-2) (Tansey et al. 2022). The presence of systemic inflammation in PD patients along with MCRs on immune cells prompted us to utilize systemic inflammatory approach with LPS. Immune responses and the integrity of the nigrostriatal dopaminergic system were assessed following NDP-MSH treatment. The role of Tregs was characterized by antibody-mediated depletion of CD25 + Tregs.

## Materials and Methods

### Study Design

The aim of the present study was to investigate the neuroprotective potential of peripherally administered NDP-MSH on the nigrostriatal dopaminergic system. We employed a subacute model of MPTP and LPS to induce systemic inflammation and dopaminergic neuronal loss. Adult C57BL/6J mice were randomly assigned to receive MPTP + LPS with or without NDP-MSH. Male gender was used due to ~ 80–100% MPTP mortality in female mice based on our observations and the literature (Jackson-Lewis and Przedborski 2007). Our first primary outcome measure was striatal DA. We used n = 11–15 mice/group. The sample size of 11 provided more than 85% power to detect a biologically meaningful 20% increase in the primary outcome measure based on our published mean ± standard deviation (SD) among WT mice for striatal DA (Chen et al. 2017b). The second primary outcome measure was nigral DA neuron counts. With n = 6–8, we also had more than 80% power to detect 20% difference based on our published mean ± SD among WT mice for this measure (Chen et al. 2017b).

NDP-MSH was injected i.p. at 400 µg/kg once daily. In healthy humans, NDP-MHS has plasma half-life of 1.3 h for s.c. injections and 1.1 h for i.v. injections(Wensink et al. 2021). It has similarly short half-life in mice though 5.1 h of serum half-life has been reported in C57BL/6 mice (Lensing et al. 2017). We performed a pharmacokinetic (PK) study. Plasma concentrations of NDP-MSH were 1785 ± 222, 810 ± 86, 118 ± 27, and 5 ± 0.5 ng/mL at 5, 15, 30, 60 min, respectively, following a single i.p. injection at 1 mg/kg in naïve C57BL/6J mice (Supplemental Table 1). Despite the relatively short half-life, accumulative neuroprotective effects of NDP-MSH have been achieved in mouse models of neuroinflammatory disease when administered i.v. at 48-hour intervals at 5 µg in 100 µl PBS (Mykicki et al. 2016) and in genetic mouse models of Alzheimer’s disease following a daily dose of NDP-MSH at 340 mg/kg for 18 weeks (Giuliani et al. 2014). The later study did not characterize MC1R engagement, BBB function, and NDP-MSH brain permeability, nor peripheral vs. CNS effects.

For primary outcome measures, the mice were sacrificed at 12 days after the last MPTP dosing. The end timepoint was based on the established subacute MPTP regimen, which induces DA deficits 7–21 days (Chen et al. 2017a; Jackson-Lewis and Przedborski 2007; Santoro et al. 2023) and a MPTP + LPS mouse model, which induces DA deficits 2 weeks post-MPTP injection (García-Domínguez et al. 2018).

### Animals

Male C57BL/6J mice (3–4 months old) (RRID:IMSR_JAX:000664) were purchased from Jackson Laboratory (Bar Harbor, ME). Mice were kept in a temperature-controlled room, with a 12-h light/dark cycle, and had free access to food and water. To assess the MC1R-dependence of effects, *Mc1r*^*e/e*^ mice (3–4 months old) (RRID:IMSR_ORNL:C3HEB-FEJ-MC1R<E-SO>) were used. *Mc1r*^*e/e*^ mice carry an inactivating mutation of *Mc1r* in a C57BL/6J background. All procedures were approved by the Institutional Animal Ethical Committee of Massachusetts General Hospital (animal protocol # 2018N000039).

### Chemicals and Treatment Paradigms

García-Domínguez et al. reported an enhanced microglia response and nigral dopaminergic cell death in an acute MPTP model following peripheral inflammation resulting from single i.p. injection of LPS at 2 mg/kg (García-Domínguez et al. 2018). We employed a subacute paradigm to inject lower doses of MPTP.HCl (20 mg/kg) and LPS (1 mg/kg) over 4 days. Mice were randomly divided into MPTP + LPS + NDP-MSH, MPTP + LPS, and control groups to receive i.p. once daily MPTP.HCl (Millipore Sigma, Cat# M0896; 20 mg/kg) or saline and LPS (Millipore Sigma, Cat# L4391; 1 mg/kg) or PBS from day 1 to day 4. NDP-MSH (Genscript, Cat# RP10658; 400 µg/kg) or PBS was injected from day 1 to day 12. Mice were tested for behavioral activities and were sacrificed thereafter on day 12.

To study the role of Tregs, animals were treated with anti-mouse CD25 monoclonal antibody (clone PC61, BioLegend, Cat# 102,059; 400 µg/mouse, RRID: AB_2813926) or isotype control (BioLegend, Cat# 401,916; 100 µg/mice) for 3 alternate days, 1 week before the start of experiment. Mice were subsequently treated with MPTP, LPS and NDP-MSH as described above. Another dose of anti-mouse CD25 monoclonal antibody or isotype control was administered 2 days before the sacrifice. The general protocol used for this treatment paradigm was deposited in protocols.io (DOI: https://www.protocols.io/view/chemicals-and-treatment-paradigms-3byl4q1kzvo5/v1).

### BBB Permeability and NDP-MSH PK Study

The integrity of BBB was measured through FITC-albumin (Millipore Sigma, Cat# A9771) leakage from vasculature into brain parenchyma as described previously (Chen et al. 2008). Mice were treated with MPTP + LPS and sacrificed after 6 and 24 h after the last dose. Briefly, mice were anaesthetized by isoflurane and perfused intracardially with heparin (100 units/kg) followed by 5 ml FITC albumin at a concentration of 5 mg/ml in PBS with a flow rate of 1.5 ml/min. Subsequently, the brain was isolated and incubated in 4% paraformaldehyde overnight at 4 degrees. The solution was changed to 30% sucrose in PBS. Coronal sections of striatum were mounted and analyzed under fluorescence microscope (Olympus BX51 microscope).

To assess NDP-MSH concentrations in plasma and brain, male mice were treated with MPTP + LPS as described above and two concentrations of NDP-MSH (400 µg/kg and 1 mg/kg) and sacrificed after 5-, 30- and 90-min. Blood samples were collected through cardiac puncture in 40 mM EDTA, and plasma was collected by centrifugation and stored at -80 °C till further analysis. The whole brain was dissected and homogenized in PBS. Proteins in brain homogenate and plasma samples were crashed with 3 volumes of methanol containing internal standard (propranolol) and centrifuged. Supernatants were analyzed by liquid chromatography/mass spectrometry (LC/MS). NDP-MSH in plasma and brain samples was detected by LC/MS through a service contract with Cyprotex, LLC, MA, USA. The general protocol used to measure BBB permeability and NDP-MSH concentrations was deposited in protocols.io (DOI: https://www.protocols.io/view/bbb-permeability-and-ndp-msh-pk-study-4r3l22p43l1y/v1).

### Open Field Test

Locomotor activity was determined at the baseline and post treatment by open field test. Briefly, the mice were placed in the plexiglass chamber (11 × 11 in with clear 8-in high walls) and were allowed to explore for a period of 10 min. The total distance travelled was measured with software Ethovision XT 9.0 (RRID:SCR_000441, https://www.noldus.com/ethovision), Noldus Information Technology, The Netherlands. The general protocol used for the open field test was deposited in protocols.io (DOI: https://www.protocols.io/view/open-field-test-n2bvj3bxnlk5/v1).

### Pole Test

Pole test was performed at the baseline and post treatment to test motor coordination and motor abnormalities that result from depletion of striatal dopamine. Mice were trained on the pole (1 cm diameter, 50 cm height) one day before the start of the experiment for 120s. Time taken by the mice to turn (T turn) and time taken to climb down (T descent) the pole were recorded (Matsuura et al. 1997; Sun et al. 2021). The general protocol used for the pole test was deposited in protocols.io (DOI: https://www.protocols.io/view/pole-test-e6nvwdq5dlmk/v1).

### Immunohistochemistry and Stereological Counting of SN Dopaminergic Neurons

Immunohistochemistry was performed on the coronal sections of SN as described previously (Cai et al. 2022). In brief, the 30 μm sections were incubated in blocking solution (10%, normal goat serum) for 1 h followed by incubating them with either of the following primary antibodies: Tyrosine Hydroxylase (TH) (1:1000) (Enzo Life Sciences, Cat# BML-SA497-0100, RRID:AB_2052772); Ionized calcium binding adaptor molecule 1 (iba1) (1:500) (Abcam Cat# ab178847, RRID:AB_2832244); Glial Fibrillary Acidic Protein (GFAP) (1:500) (BioLegend, Cat# 840001, RRID:AB_2565444) overnight at 4ºC. For peroxidase staining, sections were incubated with biotinylated secondary antibodies (Millipore Cat# OS03B-200UG, 1:2000, RRID:AB_569848; Sigma-Aldrich Cat# B7264, 1:2000, RRID:AB_258607) followed by incubating in avidin biotin complex (Vector laboratories, Cat# PK6100) and the staining was developed by incubation in 3,3′-diaminobenzidine (DAB) (Millipore Sigma, Cat# D4418). TH, iba1, and GFAP are markers for dopaminergic neurons, microglia, and astrocytes, respectively.

Stereological counting of SN TH + cells was performed to determine the total number of dopaminergic neurons in the SN as previously described (Cai et al. 2022). In brief, a complete set of coronal midbrain sections stained with TH and counterstained with Nissl was counted stereologically with Olympus BX51 microscope and Olympus CAST stereology software.

The method published by Sanchez-Guajardo et al. was referred to for analysis and classification of morphology of iba1 + microglia cells in SNpc (Sanchez-Guajardo et al. 2010). These cells can be classified according to their morphology into resting type (type A with a thin and visible cytoplasm with long and thin processes), activated type (type B with thick and short processes extending from a dense and enlarged cell body), and phagocytic type (type C with a shape resembling pseudo-amoeba, a big and dark cell body with processes). The stereological method was followed to count the cells at 40× magnification (Olympus BX51 microscope and Olympus CAST stereology software) as previously described by Dimant et al. 2013 and West et al. 1991(Dimant et al. 2013; West et al. 1991). Two midbrain sections with the central and anterior SN were analyzed per mouse.

Integrated optical density of GFAP immunoreactivity was determined by Image J (RRID: SCR_003070, https://imagej.net/) as a measurement of astrogliosis. The images were captured using ×40 objective. Two midbrain sections with the central and anterior SN were analyzed for each mouse. The general protocol used for TH cell staining was deposited in protocols.io (DOI: 10.17504/protocols.io.j8nlk4yw1g5r/v1).

### High-performance Liquid Chromatography

High-performance liquid chromatography with electrochemical detection (HPLC-ECD) was used to measure striatal dopamine levels as previously described (Chen et al. 2013; Xiao et al. 2006). Briefly, the striatum was dissected from the brain, homogenized in buffer containing perchloric acid and centrifuged at 16,000 g for 20 min followed by analysis of the supernatant through HPLC-ECD. The general protocol used for measurement of dopamine was deposited in protocols.io (DOI: 10.17504/protocols.io.dm6gpbjdplzp/v1).

Additionally striatal 1-methyl-4-phenylpyridinium (MPP+) was measured 90 min and 6 h after the last dose of MPTP. Striatum was dissected and analyzed by HPLC ultraviolet-ultraviolet (UV) detection as previously described (Chen et al. 2002).

### ELISA

Levels of IL-1β and TNF-α in plasma and brain tissue were determined by ELISA as described previously (Srivastava et al. 2020). In brief, blood samples from all the treatment groups were collected through cardiac puncture in 40 mM EDTA and plasma was collected by centrifugation. The plasma samples were immediately transferred to dry ice and then stored at -80 °C till further analysis. A small fraction of ventral midbrain tissue homogenate prepared in 1x RIPA buffer (Cell Signaling, Cat#9806) was used for analysis of IL-1β and TNF-α using mouse ELISA kits (BioLegend, Cat# 430,904; 432,604). The ELISA protocol used here was deposited in protocols.io (DOI: https://www.protocols.io/view/elisa-kqdg3xw67g25/v1).

### Flow Cytometry

To assess immune cell profile, single-cell suspension of spleen tissue was prepared according to the protocol described previously (Chauhan et al. 2018). Spleen was removed in a 35 mm petri plate with 5 ml RPMI 1640 and digested mechanically and passed through 70 μm filter screen. The cell suspension was centrifuged, and the pellet was incubated in RBC lysis buffer. The resulting cell suspension was washed in 1xPBS and blocked with Fc Block (BioLegend, Cat# 101,302, 1 µl/50 µl). The cells were incubated with MC1R (Invitrogen, Cat# PIPA521911, 1.39 µg) antibody followed by fluorophore conjugated primary antibodies for extracellular markers (BioLegend, Cat# 101235, CD11b-BV421(0.25 µg), RRID: AB_11203704; Cat# 127641, Ly6G-BV-650 (0.25 µg), RRID: AB_2565881; Cat# 128041, Ly6C-BV785 (0.125 µg), RRID: AB_2565852; Cat# 100516, CD4-APC (0.25 µg), RRID: AB_312718; Cat# 100751, CD8a-BV510 (0.5 µg), RRID: AB_2563057; Cat# 152405, CD19-PerCP-Cy5.5 (0.25 µg), RRID: AB_2629815; Cat# 102036, CD25-BV605 (0.3 µg), RRID: AB_11126977) and AF488 (Invitrogen, Cat# A11034, 1:200, RRID: AB_2576217). Zombie dye (BioLegend, Cat# 423101, 1 µl/sample) was used to differentiate between live and dead cells. Helper T cells and cytotoxic T cells were identified by CD4 + and CD8+, respectively. CD4 + CD25 + cells were used to mark Tregs. CD19 + cells were used as marker for B cells. Monocytes were identified as CD11b + Ly6G-Ly6C^high^ cells and neutrophils were marked by CD11b + Ly6C-Ly6G+. For monocytes and neutrophils, CD11b-positive cells were first extracted from the live cell subset by expansion with SSC-A, followed by Ly6C. After expansion with Ly6C and Ly6G, we excluded Ly6G-positive cells. Monocytes were identified as CD11b + Ly6G-Ly6C^high^ cells, and neutrophils were marked by CD11b + Ly6C-Ly6G+. SORP 5 Laser BD Fortessa X-20 (BD Bioscience) and FlowJo v10.7.1 (RRID: SCR_008520, https://www.flowjo.com/solutions/flowjo, Becton Dickson & Company) software was used for data acquisition. Single cell control and manual compensation were used for gating strategy. Abundance of the cell population was calculated and presented as % of the population relative to live cells from total splenocytes. FlowJo v10.7.1 (RRID: SCR_008520, https://www.flowjo.com/solutions/flowjo, Becton Dickson & Company) software was used for data analysis. The flow cytometry protocol used here was deposited in protocols.io (DOI: https://www.protocols.io/view/flow-cytometry-ewov1qm47gr2/v1).

### Statistical Analysis

Data from each experiment was presented as mean ± SEM and statistical significance was determined by One-way ANOVA with Tukey post hoc test. Two-way ANOVA with Tukey post hoc test was used to analyze neurobehavioral endpoints in open field test and pole test to compare baseline and post treatment effects. GraphPad Prism 8.3.0 (RRID: SCR_002798, http://www.graphpad.com/, GraphPad Software, San Diego, CA, USA) was used to analyze the data.

## Results

### Systemically Administered NDP-MSH Ameliorated Behavior Impairment and Protected Dopaminergic Neurotoxicity in MPTP and LPS Mouse Model of PD

**Fig. 1:**
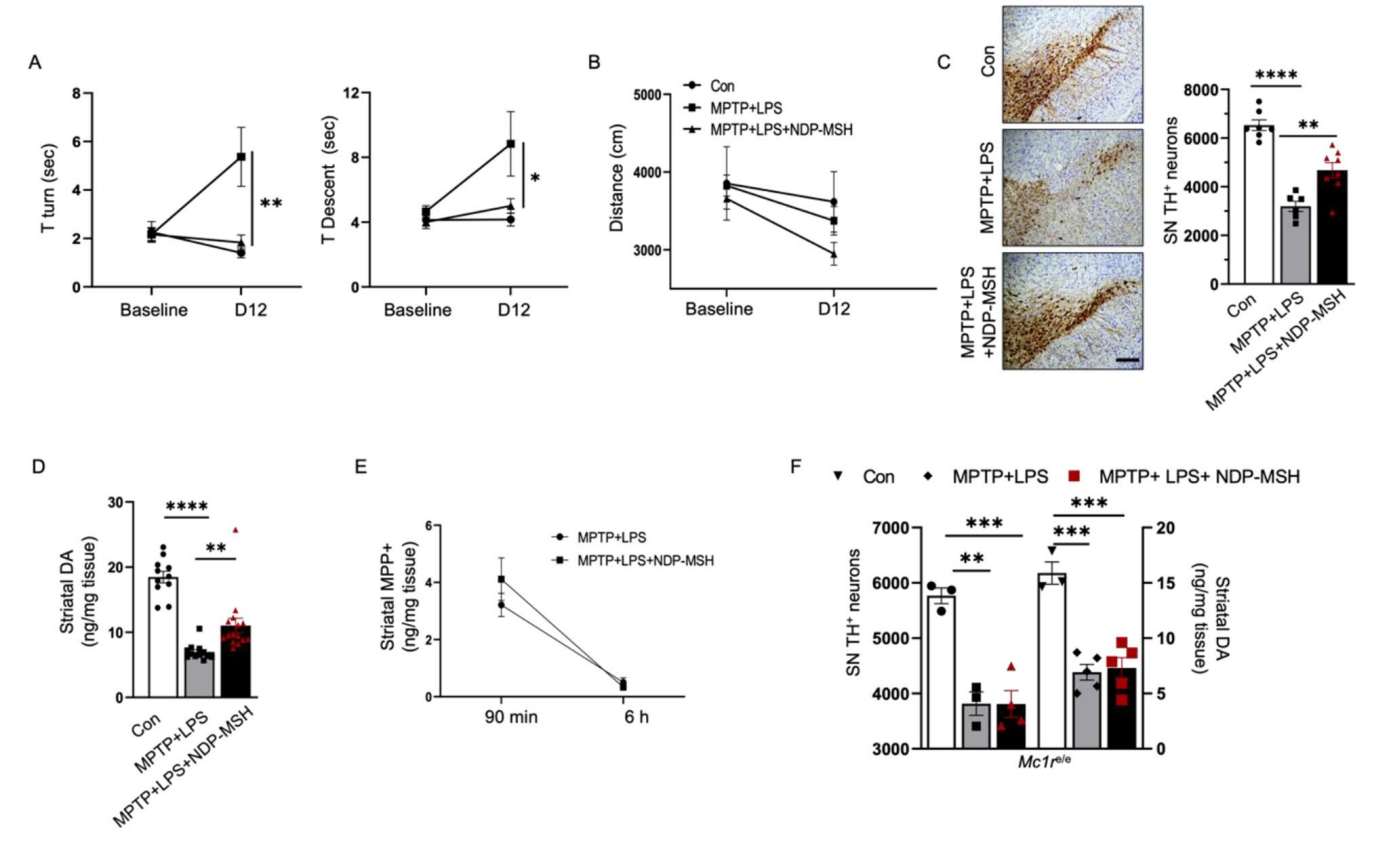
**Systemic NDP-MSH treatment protects against MPTP + LPS-induced dopaminergic neurotoxicity**. C57BL/6J mice were treated i.p. with MPTP.HCl (20 mg/kg) and LPS (1 mg/kg) or vehicle (Con) from day (D) 1 to D4 and NDP-MSH (400 µg/kg) or vehicle from D1 to D12 and sacrificed at D12. (**A**) Pole test for the time taken to turn downward (T turn) on the top of a pole and time taken to climb down (T descent) and (**B**) open field test to measure the total distance traveled. Two-way ANOVA by Tukey’s post hoc test. *p < 0.05, **p < 0.01 for MPTP + LPS vs. MPTP + LPS + NDP-MSH; n = 5–7/group. (**C**) Representative micrograph and stereological quantification of TH + cells in SN; n = 6–8/group. Scale bar, 100 μm. (**D**) Striatal dopamine content; n = 11–15/group. (**E**) Striatal MPP + levels assessed at 90 min and 6 h after treatment with MPTP + LPS with or without NDP-MSH. Two-way ANOVA followed by Tukey’s post hoc test, n = 3/group. *Mc1r*^e/e^ mice carrying non-functioning *Mc1r* were treated with the same paradigm. (**F**) Stereological quantification of TH + cells in SN (left y-axis) and striatal dopamine content (right y-axis); n = 3–5/group. One-way ANOVA followed by Tukey’s post hoc test. **p < 0.01; ***p < 0.001

We used MPTP and LPS to introduce a PD-like phenotype in the context of systemic inflammation to assess effects of NDP-MSH on behavior and the nigrostriatal dopaminergic pathway. NDP-MSH treatment in MPTP + LPS exposed mice exhibited improved behavior as they took less time to turn around and climb down in the pole test as compared with MPTP + LPS group treated with vehicle (Fig. 1A). No significant difference in distance traveled in open field was observed among the three groups (Fig. 1B).

Stereological counting of TH + neurons demonstrated that 72% SN TH + cells were preserved in MPTP + LPS mice treated with NDP-MSH, significantly higher than 49% in vehicle treated MPTP + LPS mice (Fig. 1C). Similarly, 59% striatal dopamine levels were preserved in MPTP + LPS mice treated with NDP-MSH compared with 38% in vehicle treated MPTP + LPS mice (Fig. 1D).

Following systemic administration, MPTP gets transported to the brain, metabolized into its active form MPP + that is toxic and causes death of dopaminergic neurons(Jackson-Lewis and Przedborski 2007). There was no difference in MPP + levels in the striatum between NDP-MSH vs. vehicle treated MPTP + LPS mice at 90 min and 6 h after the last dose, suggesting that NDP-MSH treatment is not associated with altered MPTP metabolism (Fig. 1E).

NDP-MSH treatment did not show any effect on TH + cell count and striatal dopamine level in *Mc1r*^*e/e*^ mice treated with MPTP + LPS, suggesting that the protective effects of NDP-MSH are also mediated through MC1R (Fig. 1F).

### BBB Permeability in MPTP + LPS mice and Penetration of NDP-MSH in Brain

**Fig. 2:**
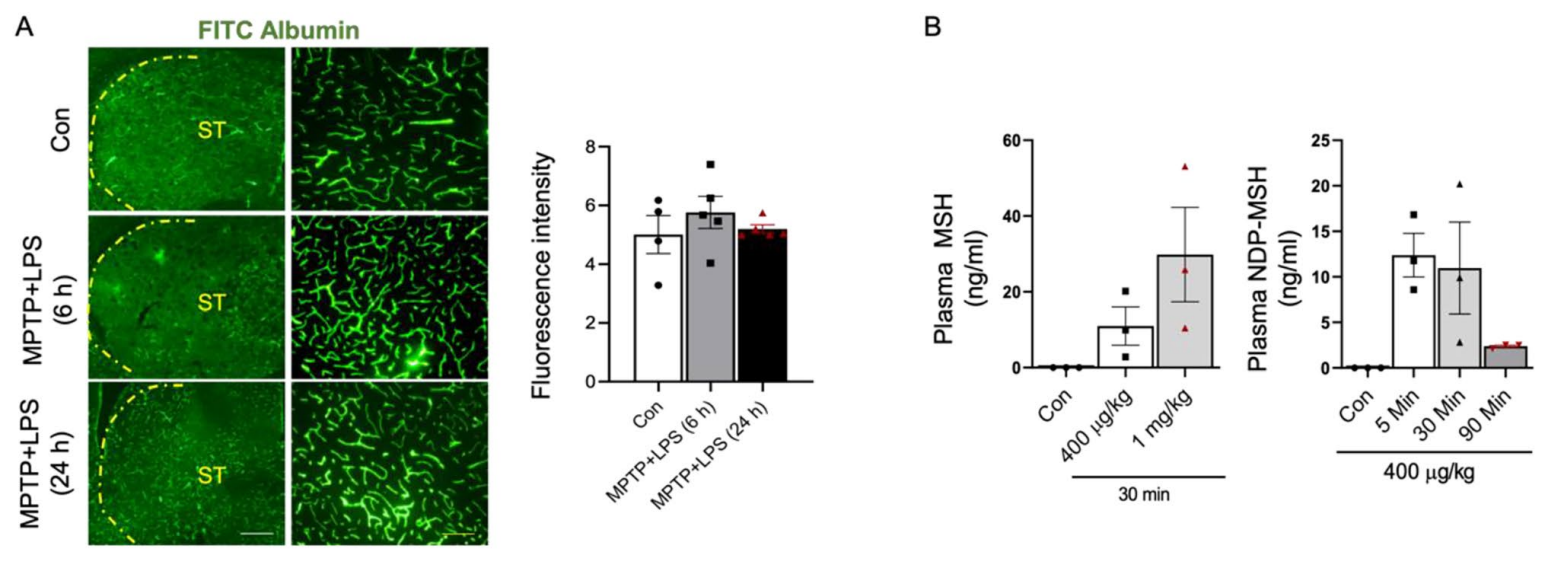
**BBB permeability in MPTP + LPS mice and pharmacokinetics of NDP-MSH**. C57BL/6 mice were treated i.p. with MPTP.HCl (20 mg/kg) and LPS (1 mg/kg) or vehicle (Con) from D1 to D4. Mice were sacrificed at 6 and 24 h after the last dose, and FITC albumin assay was conducted. (**A**) Representative micrographs of FITC albumin and quantification of fluorescence intensity in striatum. Scale bars, 30 and 100 μm. C57BL/6 male mice (3–4 months old) were treated with MPTP.HCl (20 mg/kg) + LPS (1 mg/kg) and NDP-MSH from D1 to D4 and sacrificed at 5-, 30- and 90-min. Control (Con) mice received saline injection and sacrificed at 90 min. (**B**) Levels of NDP-MSH in the plasma at different doses and different time points. Levels of NDP-MSH were below limit of detection (3.9 ng/ml) in the brain at all time points assessed. One-way ANOVA followed by Tukey’s post hoc test; n = 3/group

FITC albumin leakage assay showed that our MPTP + LPS regimen caused a slight breach in BBB permeability at 6 h but not at 24 h after the last dose (Fig. 2A). We previously reported that NDP-MSH does not cross BBB in normal C57BL/6J mice(Cai et al. 2022). To evaluate brain penetration of NDP-MSH in MTPT + LPS model, we conducted LC/MS to assess NDP-MSH concentrations in the brain and plasma at different time points. While there were time- and dose-dependent increases in NDP-MSH concentrations in the plasma (Fig. 2B), NDP-MSH in the brain was undetectable at all time points assessed. The absence of NDP-MSH in the brain could be explained by the insignificant disruption of BBB by the MPTP + LPS regimen, suggesting that the neuroprotective effects of NDP-MSH are mediated by its peripheral actions.

### NDP-MSH Reduced Inflammation in the Periphery and the Ventral Brain

**Fig. 3:**
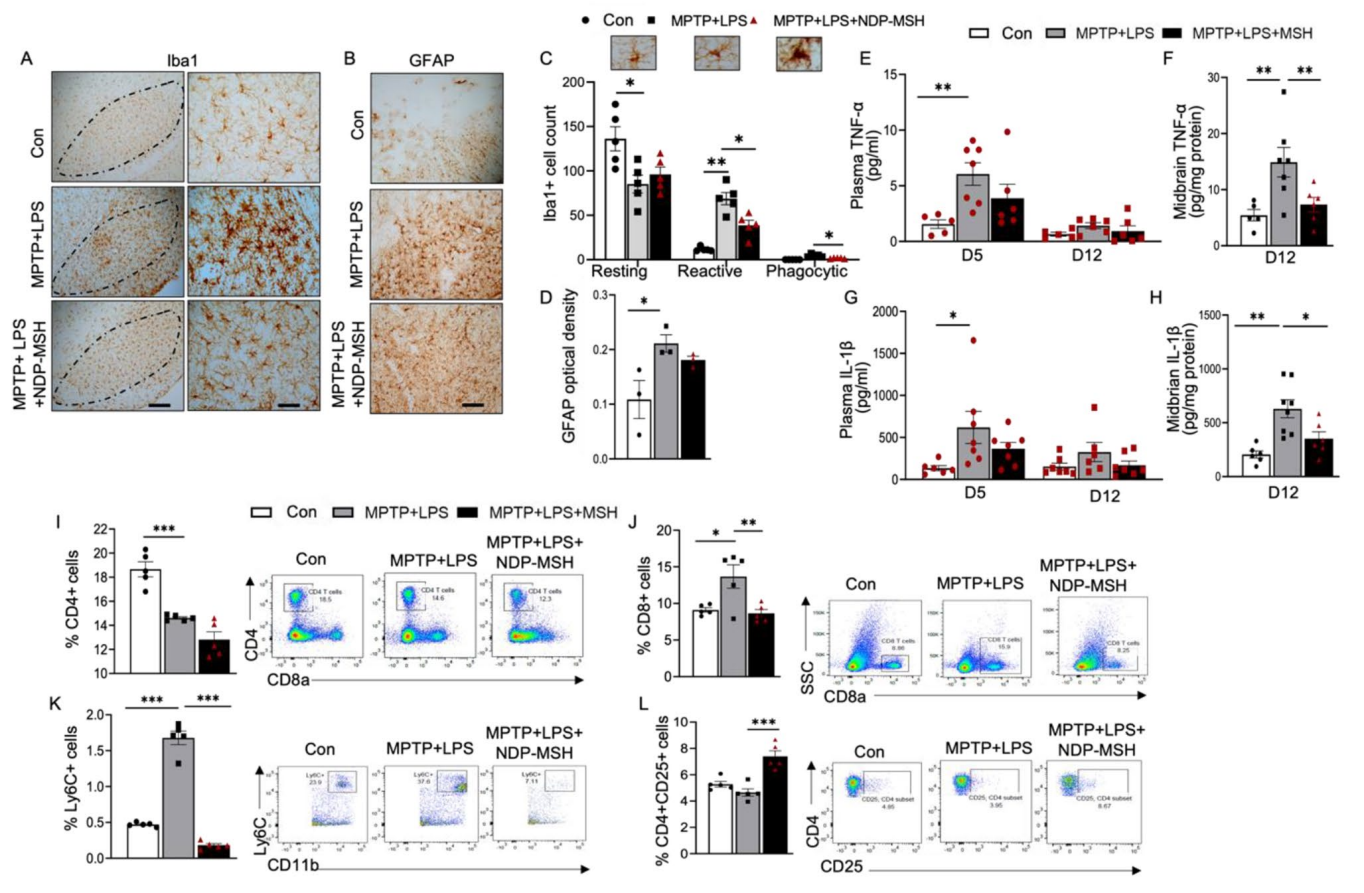
**Systemic NDP-MSH treatment reduces neuroinflammation and modulates peripheral immune responses**. C57BL/6 mice were treated i.p. with MPTP.HCl (20 mg/kg) and LPS (1 mg/kg) from D1 to D4 and NDP-MSH (400 µg/kg) or vehicle control from D1 to D12. Plasma was collected at D5 and D12. Mice were sacrificed at D12. (**A**) Cells stained positive for iba1 in the SN. Scale bars, 100 μm and 30 μm. (**B**) GFAP staining. Scale bar, 30 μm. (**C**) Morphological classification and quantification of iba1 + microglia. Two-way ANOVA followed by Tukey’s post hoc test; n = 5/group. (**D**) Quantification of integrated optical density of GFAP in the SN. One-way ANOVA followed by Tukey’s post hoc test; n = 3/group. ELISA assessment of TNF-α levels in (**E**) plasma at D5 and D12 and (**F**) ventral midbrain at D12 and IL-1β in (**G**) plasma at D5 and D12 and (**H**) ventral midbrain at D12. Two-way ANOVA followed by Tukey’s post hoc test. **p < 0.01; n = 5–7/group/time point. Flow cytometric analysis of the splenocytes showing percentages of (**I**) CD4^+^ helper T cell, (**J**) CD8^+^ cytotoxic T cells, (**K**) LY6C^+^ cell, and (**L**) CD4 + CD25^+^ Tregs. One-way ANOVA followed by Tukey’s post hoc test; *p < 0.05, **p < 0.01; ***p < 0.001; n = 4–5/group

We examined brain resident microglia and astrocytes by immunohistochemistry using iba1 and GFAP as a marker, respectively (Fig. 3A, B). iba1-positive cells were evaluated based on their morphological classification (Fig. 3C). NDP-MSH reduced microglia activation and the number of reactive and phagocytic Iba1 + microglia in the nigral region (Fig. 3A, C) but did not show effect on MPTP + LPS-induced astrogliosis (Fig. 3B, D).

Peripheral inflammation is partly responsible for activated microglia in PD (García-Domínguez et al. 2018). We assessed inflammatory cytokines and found significantly higher concentrations of TNF-α and IL1-β in plasma and ventral midbrain of MPTP + LPS mice compared with the control group (Fig. 3E, G). The increases in the levels of TNF-α and IL1-β in the brain were attenuated following treatment with NDP-MSH (Fig. 3F, H). We also observed a decrease in levels of TNF-α and IL1-β at day 5 and day 12 in plasma. Although statistically non-significant, they pointed towards attenuation of peripheral inflammation markers by NDP-MSH.

To assess the status of different immune cells in the periphery, splenocytes were stained for markers of myeloid and lymphoid cells. We assessed MC1R expression in a pilot study and observed that MC1R was expressed on a number of immune cells from the spleen in normal C57BL/6J mice under basal conditions (Supplemental Fig. 1). Exposure to MPTP + LPS caused a decrease in the percentage of CD4 + helper T cells and an increase in the percentage of CD8 + cytotoxic T cells (Fig. 3I, J), consistent with previously implicated increased percentage of circulating cytotoxic T cells in PD (Galiano-Landeira et al. 2020). NDP-MSH treatment in MPTP + LPS mice significantly increased cytotoxic CD8 + T cells percentage (Fig. 3J). Ly6C^high^ monocytes, which have also been reported to be involved in neuroinflammation (Varvel et al. 2016)were significantly increased in MPTP + LPS mice, and the change was reversed in mice treated with NDP-MSH (Fig. 3K). Additionally, despite a decrease in the percentage of total CD4 + cells, a significant increase in the percentage of Tregs was observed in NDP-MSH treated mice (Fig. 3L) compared to the untreated MPTP + LPS group. These results indicated possible involvement of Tregs, at least in part, in NDP-MSH impact on inflammation and neuroprotection.

### Depletion of Treg Cells Limited the Neuroprotective Effect of NDP-MSH

**Fig. 4:**
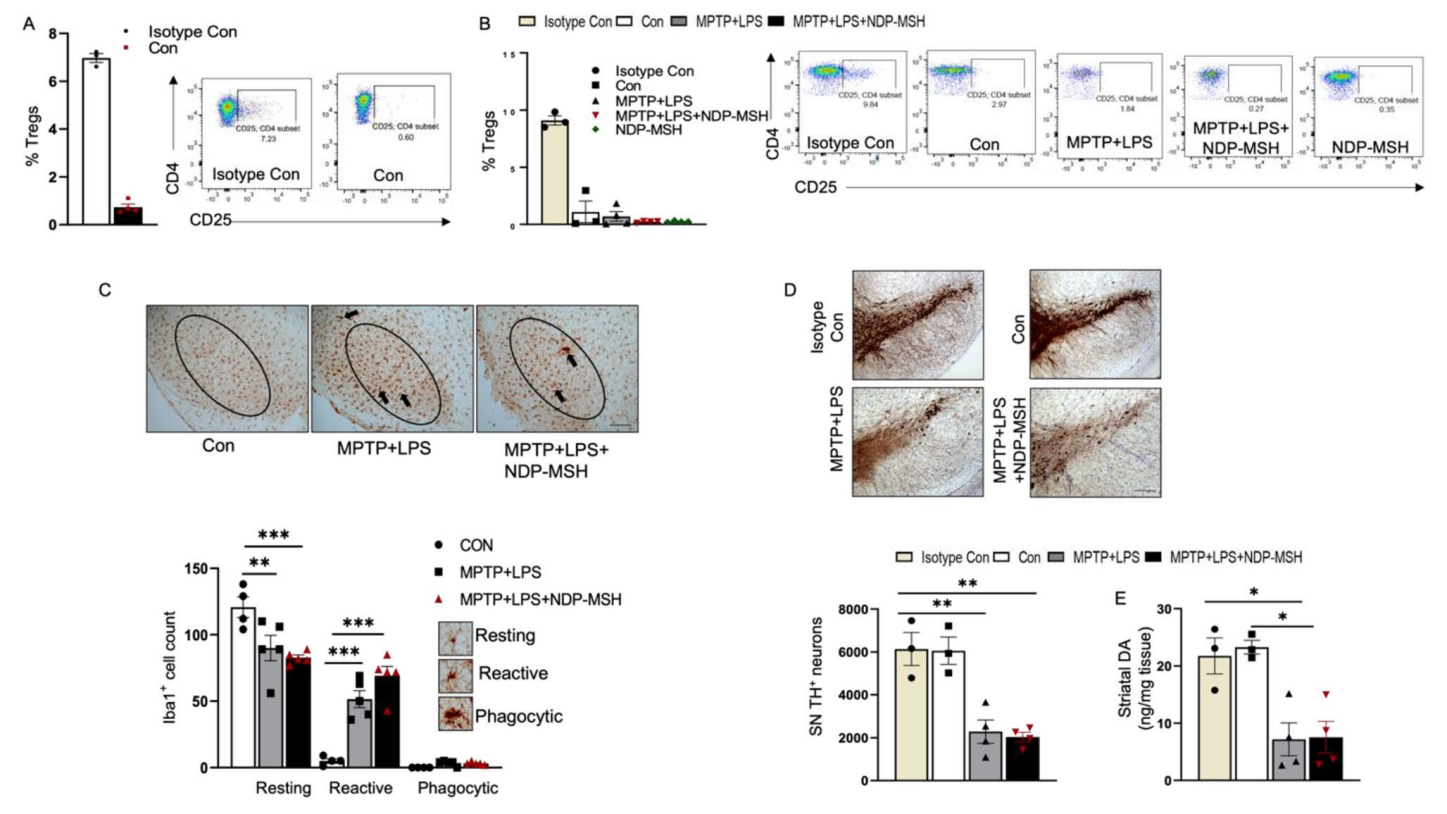
**Depletion of Tregs abolishes neuroprotective effect of NDP-MSH**. (**A**) C57BL/6 mice were pre-injected with PC61/CD25 antibody or isotype control. Mice were then injected with MPTP.HCl (20 mg/kg) + LPS (1 mg/kg) or vehicle (Con) and treated with NDP-MSH (400 µg/kg) or vehicle. Mice were sacrificed, and flow cytometry was carried out to assess the percentage of Treg cells in the spleen (**A**) before random grouping and (**B**) at the end of the treatment paradigm. (**C**) Representative micrograph staining for iba1 and morphological classification and quantification of iba1 + microglia in SN. scale bar 100 μm; n = 4/group. Two-way ANOVA followed by Tukey’s post hoc test; **p < 0.01; ***p < 0.001. (**D**) Representative micrograph of TH + staining and stereological quantification of TH + cells in SN. Scale bar, 100 μm; n = 3–4/group. One-way ANOVA followed by Tukey’s post hoc test. **p < 0.01. (**E**) Striatal dopamine content; n = 3–4/group. One-way ANOVA followed by Tukey’s post hoc test. *p < 0.05. Representative results from 2–3 independent experiments, which were conducted using preserved samples or fresh samples and were not pooled

Mykicki et al. have shown induction of Treg cells following treatment with NDP-MSH in EAE (Mykicki et al. 2016). In the present study we observed a significant increase in the percentage of Tregs following administration of NDP-MSH in MPTP + LPS mice (Fig. 3L). To evaluate whether Tregs may be involved in NDP-MSH neuroprotection, we used PC61/CD25 antibody to deplete Tregs in mice. Mice were pretreated with the antibody, and then randomly grouped and treated with MPTP + LPS and NDP-MSH as described above. Depletion of Treg cells was confirmed before the randomization and at the end of the experiment among the treatment groups (Fig. 4A, B). NDP-MSH failed to attenuate activation of iba1 + microglia in MPTP + LPS mice lacking Tregs (Fig. 4C).

MPTP + LPS treated with vehicle resulted in 63% and 63% TH neuron loss and 70% and 68% dopamine reduction relative to control in Treg depleted mice and isotype control, respectively, while NDP-MSH group showed 67% and 67% TH neuron loss and 68% and 66% dopamine reduction relative to control in Treg depleted mice and isotype control, respectively. We did not observe any protective effects on TH + cell count and striatal dopamine levels in Treg depleted mice treated with MPTP + LPS and NDP-MSH (Fig. 4D, E), suggesting that neuroprotective effects of NDP-MSH may be dependent on peripheral Tregs.

## Discussion

MC1R is found in both the peripheral and CNS, implying that both the peripheral and central forms of MC1R might potentially impact dopaminergic neurons in Parkinson’s disease. We previously reported MC1R-dependent neuroprotection of locally injected NDP-MSH in brain against αSyn dopaminergic neurotoxicity(Cai et al. 2022). The present study demonstrates that peripherally administered NDP-MSH protects dopaminergic neurons in a combined MPTP and LPS mouse model of PD. Intraperitoneal NDP-MSH improved behavioral performance in the pole test, and attenuated loss of nigral TH + cells and striatal dopamine induced by MPTP + LPS. The dopaminergic neuroprotective effects are associated with significantly tempered microglia activation and reduced pro-inflammatory cytokines.

Our findings add to the growing evidence of the beneficial neuroprotective influence of peripherally administered NDP-MSH in models of various neurological disorders (Giuliani et al. 2014; Mykicki et al. 2016; Gatti et al. 2012). The levels of circulating α-MSH reportedly decline in patients with subarachnoid hemorrhage (Gatti et al. 2012). Fu et al. reported ameliorative effects of NDP-MSH on oxidative stress and apoptosis in the affected neurons in mouse models of intracerebral hemorrhage (Fu et al. 2020). Mykicki et al. reported that NDP-MSH injected intravenously ameliorates neuroinflammation and EAE progression via signaling through orphan nuclear 4a receptor (Nr4a) (Mykicki et al. 2016). Although NDP-MSH can bind to other MCRs, it has highest affinity to MC1R (Haskell-Luevano et al. 2001). Using *Mc1r*^*e*/e^ mice, Mykicki et al. further demonstrated that the beneficial effect of NDP-MSH is mediated by MC1R (Mykicki et al. 2016). Wu et al. reported similar MC1R mediated attenuation of neuroinflammation via CREB/Nr4a1/NF-κB pathway following i.p. injection of NDP-MSH (Wu et al. 2019). Our study similarly demonstrated the requirement of MC1R for NDP-MSH dopaminergic neuron protection. Together with previous studies from our group and others, our findings support the role of MC1R in neuroinflammation and dopaminergic neurodegeneration in PD. However, the involvement of other MCRs cannot be excluded. In a transgenic mouse model of AD, the most common age-related neurodegenerative disease, NDP-MSH intraperitoneal induced neurogenesis and cognitive recovery, and an MC4R antagonist abolished the beneficial effects of NDP-MSH (Giuliani et al. 2014). Further studies are needed to elucidate possible involvement of other MCRs in mediating NDP-MSH mediated dopaminergic neuroprotection in models of PD.

NDP-MSH is a relatively large peptide with no indications of brain penetrability in either normal mice (Cai et al. 2022) or in the MPTP + LPS model of PD. In the abovementioned studies using animal models of hemorrhage and EAE, which are known to have disrupted BBB, peripherally injected NDP-MSH restored BBB integrity (Mykicki et al. 2016; Wu et al. 2019; Gatti et al. 2012). A disruption in BBB permeability has been reported in postmortem studies in PD patients (Gray and Woulfe 2015) and in models of PD (Chen et al. 2008; Brochard et al. 2008). The most common causative factors for this disruption have been proposed to be oxidative stress and neuroinflammation. A study with an MPTP mouse model of PD reported transient leakage of serum proteins and immune cells from the brain vasculature due to increased BBB permeability (Brochard et al. 2008). We observed extravasation of FITC-albumin at 6 h after last dosing of MPTP + LPS but not at 24 hours’ time point. Furthermore, NDP-MSH was not detectable at any of the timepoints we assessed. These results suggest that NDP-MSH likely exerted neuroprotective effects through its peripheral actions in our present model system. The peripheral targets of NDP-MSH include specific immune cell populations. NDP-MSH reversed the MPTP + LPS-induced increases in percentages of monocytes and cytotoxic CD8 + T cells. Studies have reported increased infiltration of cytotoxic CD8 + T cells and little or no change in CD4 + T cells in PD patients and in MPTP models (Galiano-Landeira et al. 2020; Schröder et al. 2018) of PD.

Monocytes have also been implicated in PD pathogenesis. PD blood monocyte populations have more proliferative capacity compared to healthy controls (Nissen et al. 2019). Ly6C^high^, the proinflammatory subset of monocytes, are increased peripherally in αSyn transgenic mice (Grozdanov et al. 2019), and LPS animal models also exhibit increased infiltration of Ly6C^high^ monocytes which consequently leads to increased levels of TNF-α and IL-1β (Kratofil et al. 2017; Lessard et al. 2017; Zheng et al. 2016). We found increased levels of TNF-α and IL-1β in MPTP + LPS mice, and also the ability of NDP-MSH to ameliorate the changes in cytokine levels in the ventral midbrain. TNF-α and IL-1β are among the most studied cytokines in the context of PD (Tansey and Goldberg 2010). Elevated TNF-α levels in animal models have been associated with neuroinflammation and the degeneration of dopaminergic neurons (Leal et al. 2013). Increased IL-1β has also been demonstrated in PD animal models. It is involved in neuroinflammatory processes and may contribute to the progression of neurodegeneration (Mogi et al. 1994, 1996; Leal et al. 2013) Although findings from clinical studies have been variable, elevated levels of TNF-α and IL-1β have been reported in CSF or serum of PD patients (Blum-Degena et al. 1995; Tansey and Goldberg 2010). These studies suggest the potential involvement of TNF and IL-1β in PD.

Tregs play a critical role in regulating immune tolerance and homeostasis. Treg dysregulation has been implicated in PD, and adoptive transfer of Tregs is neuroprotective in MPTP models of PD (Huang et al. 2020). MC1R signaling reportedly triggers the expansion of Treg by acting on dendritic cells (Nasti and Timares 2015). Auriemma et al. showed that MC1R activated tolerogenic dendritic cells that stimulated and expanded functional CD4 + CD25 + Foxp3 + Tregs (Auriemma et al. 2012). NDP-MSH has also been shown to induce functional Tregs in EAE models (Mykicki et al. 2016). In this study we found elevated levels of Tregs in response to NDP-MSH in MPTP + LPS treated mice. Tregs-mediated neuroprotection is reportedly a result of increased neurotrophins, reduced proinflammatory molecules, cytokines, and oxidative stress, and induced apoptosis in the M1 state of microglia (Park et al. 2022; Gendelman and Appel 2011). Upon depletion of these cells, NDP-MSH neither abrogated microglia activation nor showed protective dopaminergic neuroprotection in the MPTP + LPS model of PD. These findings suggest that Tregs may play a role mediating the neuroprotective effects of NDP-MSH. However, it is important to consider alternative interpretations, such as the possibility that the severity of the lesion exceeds the protective capacity. It is also possible that Treg depletion changes MPTP sensitivity. Further study could employ Tregs adoptive transfer to investigate specificity of Tregs in NDP-MSH neuroprotection. Future studies are also needed to address how Treg function or marker expression may alter with NDP-MSH treatment and how Tregs may directly or indirectly mediate the dopaminergic neuroprotective effects of peripherally administered NDP-MSH.

Our study has limitations. First, our broader approach for Treg characterization examined the entire CD25-expressing populations, which include Tregs but also potentially activated effector T cells. We did not include more specific intracellular marker FOXP3. In addition, we did not analyze CD8+, B cells, and monocytes in detail. CD8 + cells particularly appear to show the strongest MC1R signal. MCR signaling has been reported to transform CD4 + T effector cells into CD4 + CD25 + Tregs (Taylor and Namba 2001)and reactive CD8 + cells in tolerogenic type in murine contact dermatitis (Andersen et al. 2017; Loser et al. 2010). More studies should be conducted to explore CD8 + and other immune cell populations in the MPTP + LPS model and their responses to NDP-MSH. Future studies should also aim for fuller characterization of the systemic immune profile changes to include myeloid-derived suppressor cells, an anti-inflammatory and immunosuppressive subset of cells, and to include peripheral blood to determine alterations in the circulating immune phenotype in response to NDP-MSH. Secondly, we used iba1 for microglial morphology. Incorporating other complementary markers such as CD68, MHC-II, TMEM119 would provide a more comprehensive characterization of microglial phenotype and function (Jurga et al. 2020). Although we assessed infiltration of the immune cells into the brain the results were not clear due to low cell viability. Future studies should examine myeloid cell polarization to explicitly distinguish between resident and infiltrating myeloid cells in the midbrain. Third, although our data indicates the roles of cytokines, specifically TNF-α and IL-1β in mediating peripheral and CNS inflammation, how the peripheral immune responses to MDP-MSH improve CNS inflammation and dopaminergic integrity will need further investigation to better explain the link between peripheral and central effects of MDP-MSH. Lastly, we employed the concurrent treatment paradigm. Future studies should further explore treatment after lesion onset, which could provide insight into the potential for NDP-MSH to ameliorate disease progression and help assess the therapeutic potential of peripherally administered NDP-MSH as a disease-modifying intervention. Additionally, a pretreatment approach could be employed to examine whether pre-exposure to NDP-MSH has a preventive effect on the development of dopamine deficits beyond what we observed in the present study.

## Conclusion

The present study demonstrates that NDP-MSH protects nigrostriatal dopaminergic neurons in the MPTP + LPS model of PD. The neuroprotective effects of NDP-MSH are likely mediated by its peripheral actions and are MC1R dependent. In addition, NDP-MSH protects against MPTP + LPS-induced immune dysregulation and inflammation. Tregs may be necessary in the protective effect of NDP-MSH.

Together with previous studies from our group and others, our study supports the role of peripheral or systemic MC1R and the peripheral immune system, particularly Tregs, in the pathophysiology of PD. It also supports peripheral MC1R activation as a therapeutic strategy for PD. NDP-MSH is an approved drug currently used to prevent skin damage from sun exposure in people with erythropoietic protoporphyria (Langendonk et al. 2015). Our demonstration that the peripheral actions of NDP-MSH can be sufficient to protect dopaminergic neurons supports a rationale for repurposing NDP-MSH as a disease-modifying agent for PD.

.

## Data Availability

The datasets used and/or analyzed during the current study are available at open-access repository through zenodo.com; DOI: 10.5281/zenodo.7383131.

## List of Abbreviations

MCRs: Melanocortin receptors
MC1R: Melanocortin 1 receptor
α-MSH: Alpha-melanocyte stimulating hormone
SN: Substantia nigra
αSyn: Alpha-synuclein
MPTP: 1-methyl-4-phenyl-1,2,3,6-tetrahydropyridine
AD: Alzheimer’s disease
BBB: Blood brain barrier
EAE: Experimental autoimmune encephalomyelitis
LPS: Lipopolysaccharide
TH: Tyrosine Hydroxylase
GFAP: Glial Fibrillary Acidic Protein
HPLC-ECD: High-performance liquid chromatography with electrochemical detection
Nr4a: Nuclear 4a receptor
